# Supervised machine learning to validate a novel scoring system for the prediction of disease remission of functional pituitary adenomas following transsphenoidal surgery

**DOI:** 10.1038/s41598-023-42157-3

**Published:** 2023-09-16

**Authors:** Chase McKevitt, Ellie Gabriel, Lina Marenco-Hillembrand, Andrea Otamendi-Lopez, Suren Jeevaratnam, Joao Paulo Almeida, Susan Samson, Kaisorn L. Chaichana

**Affiliations:** 1 Department of Neurological Surgery, Mayo Clinic, 4500 San Pablo Road, Jacksonville, FL 32224 USA; 2 Division of Endocrinology, Diabetes and Metabolism, Department of Medicine, Mayo Clinic, 4500 San Pablo Road, Jacksonville, FL 32224 USA

**Keywords:** CNS cancer, CNS cancer, Surgical oncology

## Abstract

Functional pituitary adenomas (FPAs) are associated with hormonal hypersecretion resulting in systemic endocrinopathies and increased mortality. The heterogenous composition of the FPA population has made modeling predictive factors of postoperative disease remission a challenge. Here, we aim to define a novel scoring system predictive of disease remission following transsphenoidal surgery (TSS) for FPAs and validate our process using supervised machine learning (SML). 392 patients with FPAs treated at one of the three Mayo Clinic campuses were retrospectively reviewed. Variables found significant on multivariate analysis were incorporated into our novel Pit-SCHEME score. The Pit-SCHEME score with a cut-off value ≥ 6 achieved a sensitivity of 86% and positive likelihood ratio of 2.88. In SML models, without the Pit-SCHEME score, the k-nearest neighbor (KNN) model achieved the highest accuracy at 75.6%. An increase in model sensitivity was achieved with inclusion of the Pit-SCHEME score with the linear discriminant analysis (LDA) model achieving an accuracy of 86.9%, which suggests the Pit-SCHEME score is the variable of most importance for prediction of postoperative disease remission. Ultimately, these results support the potential clinical utility of the Pit-SCHEME score and its prospective future for aiding in the perioperative decision making in patients with FPAs.

## Introduction

Pituitary adenomas are benign tumors arising from the adenohypophysis^1^. Functional pituitary adenomas (FPAs) are associated with systemic endocrinopathies due to hypersecretion of specific anterior pituitary hormones including andrenocortiotrophic hormone (ACTH), growth hormone (GH), prolactin (PRL), and thyroid stimulating hormone (TSH)^2^. For the majority of FPAs, transphenoidal surgery (TSS) is considered first-line treatment with a goal of maximal safe resection and postoperative disease remission.^2^ While dopamine agonist (DA) therapy is often the first line management in patients with functional lactotrophic adenomas, a minority of patients will undergo TSS due to adverse effects from or treatment resistance to DA therapy^2^.

Historically, the heterogenous composition of the FPA population has made modeling predictive factors for postoperative disease remission in patients with FPAs a difficult challenge. Here, we aim to overcome this challenge by utilizing a variety of statistical modeling techniques including ROC analysis, multivariate regression models and supervised machine learning (SML) to create and validate a novel scoring system for the prediction of postoperative disease remission in patients with a FPA.

## Methods

### Subject selection

**Figure 1:**
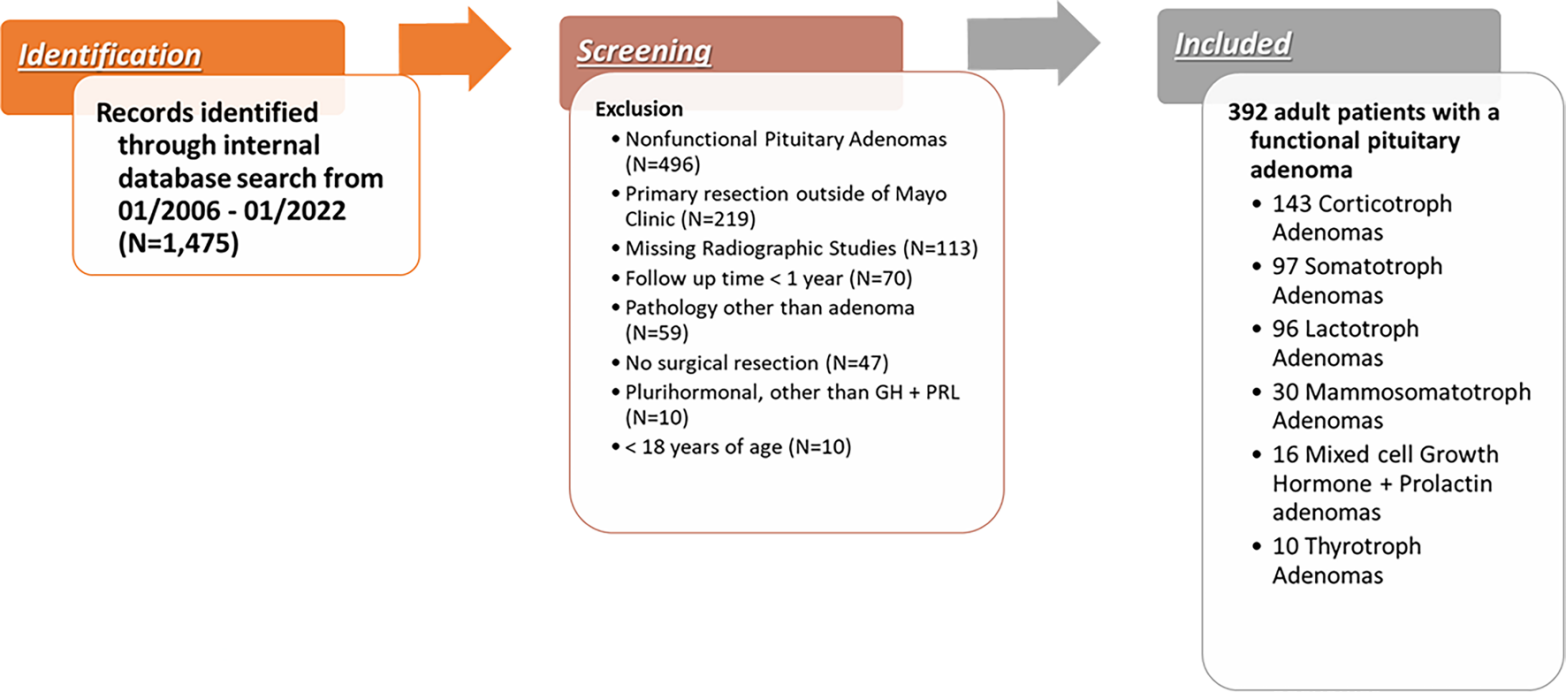
Flow chart outlining the subject selection of 392 Patients with a functional pituitary adenoma.

A retrospective study was conducted on 392 adult (≥ 18 years old) patients who underwent primary TSS for a FPA, including somatotrophic (SA), corticotrophic (CA), thyrotrophic (TA), lactotrophic (LA), mammosomatotrophic (MSA), and mixed cell GH/PRL adenomas. Figure 1 is a flowchart overviewing the subject selection of our cohort. Initially, 1475 patients with a presumed diagnosis of pituitary adenoma who underwent treatment at one of our three Mayo Clinic campuses between January 2006 and January 2022 were reviewed. The inclusion criteria were the following: patients with FPAs who underwent primary TSS resection at one of the three Mayo Clinic campuses, pathology reports with appropriate immunohistochemical staining according to the 2022 World Health Organization (WHO) classification of pituitary neuroendocrine tumors, follow up period of at least one year, and appropriate preoperative and postoperative magnetic resonance imaging (MRI)^1^.

### Preoperative evaluation of pituitary hormone status and criteria for disease remission

Confirmation of preoperative pituitary hypersecretion was based on current consensus guidelines^3,4^. For diagnosis of ACTH-dependent hypercortisolism (Cushing’s disease (CD)) the criteria was the following: (1) two unequivocally abnormal results of any of the three first-line tests including late night salivary cortisol (at least two measurements), 24-h urinary cortisol, or the overnight 1 mg dexamethasone suppression test (DST), (2) and ACTH-dependence established with an ACTH with a level > 20 pg/mL^3,4^. If indicted, inferior petrosal sinus sampling and/or an 8 mg DST was used to confirm CD^4,5^. The diagnosis for acromegaly was made with an elevated serum Insulin-like Growth Factor-1 (IGF-1) and clinical manifestations suggestive of acromegaly^4^. Hyperprolactinemia due to a FPA was based on abnormally elevated serum PRL levels with a sellar mass identified on MRI^4^. Finally, the diagnosis of central hyperthyroidism was based on inappropriately normal or high serum TSH in conjunction with elevated serum free T4 and T3 concentrations and a sellar mass on MRI^4^.

Biochemical remission for CAs was defined as normalization of plasma cortisol levels^5,6^. Biochemical remission for SAs was defined as a random GH ≤ 1.0 ng/mL with a normal IGF-1 for age^7^. Remission for LAs was defined as sex specific normalization of PRL levels^2^. Remission for TAs was defined as normalization of thyroid hormone levels^2^. Finally for both MSA and mixed cell GH/PRL adenoma remission was defined as normalization of both PRL and IGF-1^8^. At 6 months following surgery, all patients who achieved biochemical remission with the absense of additional postoperative treatment (radiation therapy, repeat surgery, or medical therapy) were considered to be in disease remission.

### Variables

All variables were retrospectively obtained from electronic chart review. Demographic information included age, sex and follow-up duration. For LAs a pre-and postoperative POD1 PRL index (PRL value/sex specific upper range of normal PRL) was tabulated. For all GH-secreting adenomas (SA, MSA, Mixed GH/PRL adenomas) preoperative and 3 month postoperative (POM3) IGF-1 indices (IGF-1 value/sex and age specific upper range of normal IGF) were obtained in addition to POD1 serum basal GH levels. Preoperative ACTH, 1 mg DST, POD1 serum ACTH, and POD1 morning serum cortisol levels were documented for CAs. For TAs, pre-and postoperative TSH, free T4, T3 levels were recored. For LAs, preoperative dopamine agonist use was documented. All radiographic variables were obtained from preoperative MRI with and without gandolinum and included largest diameter (in mm), Knosp grade, suprasellar extension, anterior fossa extension, and posterior fossa extension^9^. The operative visual technique was documented as either endoscopic or microscopic. Resection technique was either a piecemeal or pseudocapsular resection based on the surgical operative note. Surgical outcomes were classified as gross-total resection (GTR) or subtotal-resection (STR) as defined by lack of resuidal adenoma on three month postoperative MRI. Postoperative complications documented included new postoperative cerebrospinal fluid (CSF) leak, diabetes insipidus (DI), new onset hypopituitarism (defined as ≥ 1 deficient hypothalamic-pituitary axis), vascular injury, and cranial nerve injury.

### Statistical analysis

Statistically analyses were conducted using IBM SPSS version 27 (IBM Corp.) and R4.2.1. For all statistical analyses, a p-value < 0.05 was considered significant. Receiver operative characteric (ROC) curves were used to define predictive biochemical cut-off values. A biochemical cut-off value of at least 80% sensitivity for disease remission was selected for all statistically significant ROC curves. Univariate analyses for the evaluation of variables associated with postoperative disease remission included including Chi-square, independent T, Mann–Whitney U tests. All variables found to associated with postoperative disease remission in univariate analysis were then included in a multivariate binary logistic regression model. An identical modeling process was implemented for adenoma subgroup analysis. Factors deemed to be predictive of disease remission on multivariate modeling were incorporated into our novel Pit-SCHEME score. Tolerance and variance inflation factor (VIF) were calculated to evaluate for multicollinearity between the Pit-SCHEME score and its independent subcomponents. Thresholds for multicollinearity were set at a tolerance < 0.1 and VIF > 10.

Supervised machine learning (SML) models were implemented to compare the accuracy of the Pit-SCHEME score against the multivariate model. Six supervised machine learning algorithms spanning linear, decision tree, and Bayesian methods were trained and tested as binary classifiers to predict disease remission in FPA patients who underwent transsphenoidal surgery: (1) linear discriminant analysis (LDA), (2) random forest (RF), (3) classification and regression tree (CART), (4) k-nearest neighbor (kNN), (5) naïve Bayes, and (6) support vector machines (SVM) with radial kernel. Six multivariate predictors and the Pit-SCHEME score were used as variables. Grid search and ten-fold cross-validation were performed for each model to select the best performing hyperparameters. The models were trained and tested on unseen data using an 80/20 (308/76 patient) pseudo-random split. Data preprocessing included rescaling Pit-SCHEME score to between 0 and 10. Models were evaluated using prediction accuracy: accuracy = (true positives (TP) + true negatives (TN)/(TP + false positives + TN + falnse negatives). ROC curves were generated and area under the curve (AUC) was calculated to further evaluate the models. Variable importance for the best-performing models are reported to improve interpretability of the novel score and asses the clinical relevance of the patient characteristics. To compare model performance, the Wilcoxon signed rank test was used to determine statistically significant differences n predictions between models^10^. The caret R package was used for model training, hyperparameter tuning, and testing^11^.

### Ethical considerations

The Mayo Clinic Institional Review Board approved the study (ID 16-00946) and waived the requirement for obtainment of informed consent. All patient identifiers have been removed and individual patient information will not be made public. The study conformed to the principles outlined in the Delcation of Helsinki.

## Results

### Population characteristics

Demographic, radiographic, surgerical, and endocrinological information is summarized in the Supplementary Table 1. CA (36.5%) were the most prevalent subtype followed by SA (24.7%) and LA (24.5%). The mean age of diagnosis was 44.89 (Standard Deviation (SD): ± 15.15) years with 269 females (68.6%) and 123 males (31.4%). In terms of sex distrubtion for particular histological subgroups: for LAs 48 females (45.8%) and 52 males (54.2%), for CAs 119 females (83.2%) and 24 males (16.8%), for SAs 63 females (64.9%) and 32 males (35.1%), for MSAs 21 females (70%) and 9 males (30%), and for mixed cell GH/PRL adenomas 6 females (37.5%) and 10 males (62.5%). Macroadenomas comprised 248 (63.3%) of the cohort. 286 (73%) patients had a low Knosp grade (0–2), and 106 (27.0%) with a high Knosp grade (> 3) suggestive of cavernous sinus invasion (CSI). Extension into the suprasellar, anterior fossa, and posterior fossa were seen in 145 (37.0%), 30 (7.7%), and 7 (1.8%) patients, respectively. All patients underwent TSS with 259 (66.1%) with an endoscopic technique. A GTR was achieved in 284 (72.4%) patients. In terms of postoperative complications, 24 (6.1%) patients required repair of a CSF leak, 44 (11.2%) developed hypopituitarism, and 66 (16.9%) experienced DI.

**Figure 2:**
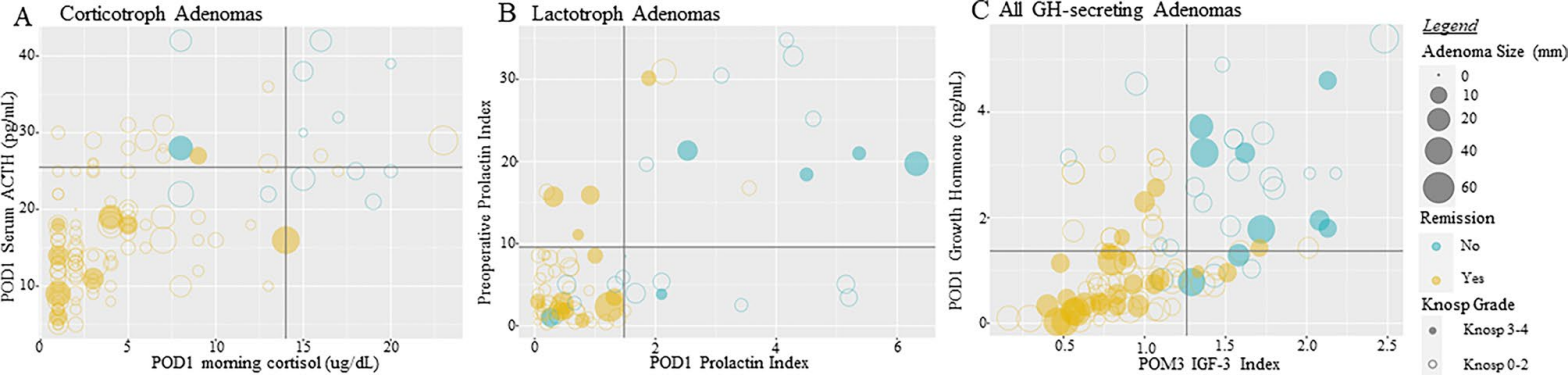
Relationship between biochemical levels, adenoma size, and Knosp grade with postoperative remission. Adenoma size is represented by the magnitude of the circle. A darker shaded circle represents a high Knosp grade (> 3). Black lines represent predictive biochemical cut-off values. (**A**) Relationship among corticotrophic adenomas. (**B**) Relationship among lactotrophic adenomas. (**C**) Relationship among all GH-secreting adenomas.

Biochemical values are highlighted in Supplementary Table 1 and Fig. 2. For CAs, the median preoperative ACTH value was 74.00 pg/mL (Interquartile Range (IQR): 45.00–105.00 pg/mL) and mean serum cortisol following 1 mg DST was 14.27 ug/dL (SD: ± 8.41 ng/dL). Postoperatively, the median POD1 ACTH value was 21.50 pg/mL (IQR: 13.00–28.00 pg/mL) and the median POD1 serum morning cortisol level was 5.00 ug/dL (IQR: 2.10–13.00 ug/dL). 69.8% (N = 68) of patients with LAs received preoperative dopamine agonist therapy. For LAs, the median preoperative PRL index was 5.05 (IQR: 2.00–19.43) and median POD1 PRL index was 0.91 (IQR: 0.303–3.700). For all GH-secreting adenomas the perioperative somatotrophic hormone values were the following: mean preoperative IGF-1 index of 2.78 (SD: ± 1.04), median POD1 GH of 1.43 ng/mL (IQR: 0.7250–3.215 ng/mL), and a median POM3 IGF-1 index of 1.09 (IQR: 0.780–1.700).

### Defining predictive preoperative and postoperative biochemical lab values

**Figure 3:**
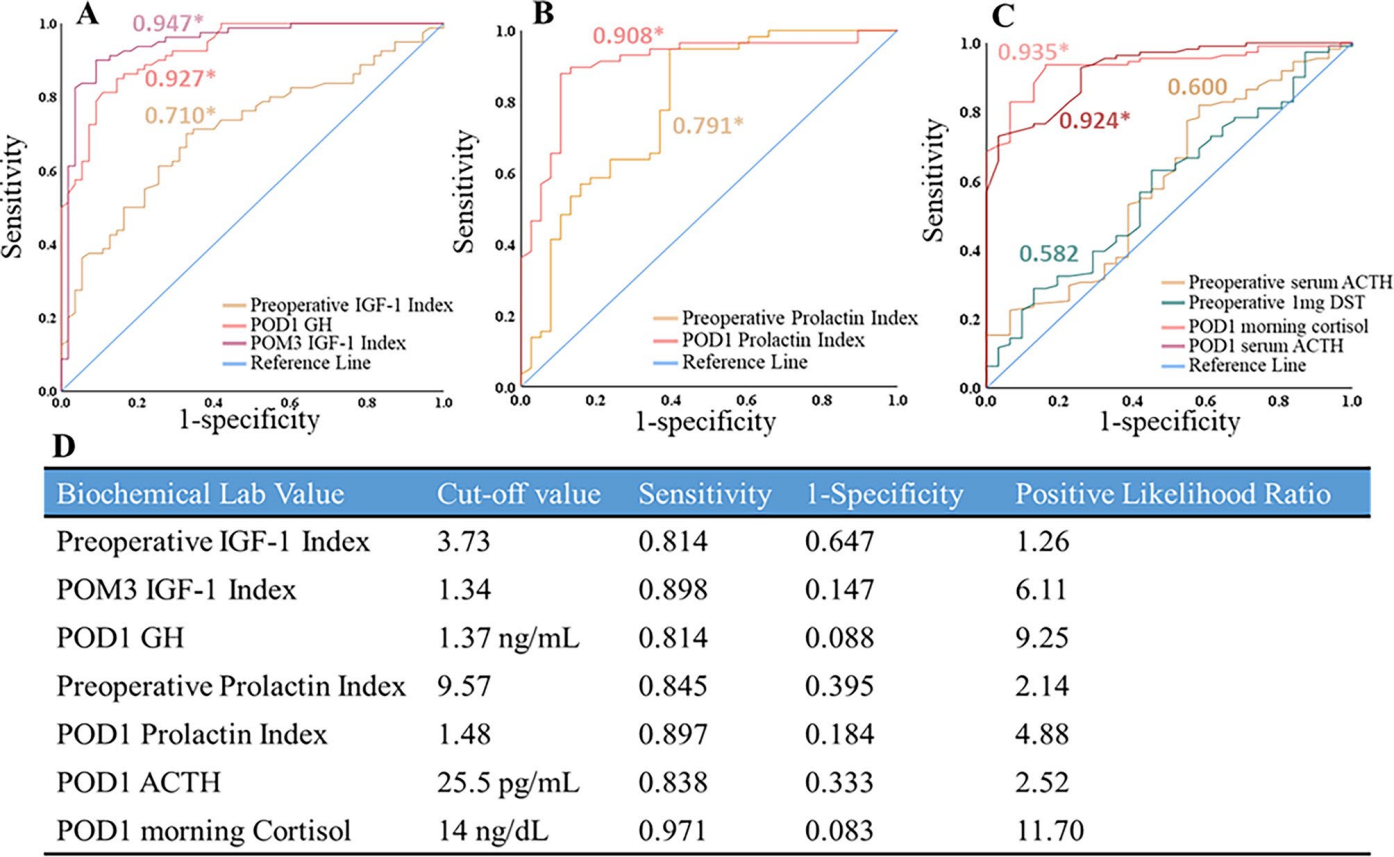
Using ROC curves to determine predictive biochemical cut-off values. Each ROC curve is labeled with their corresponding AUC. AUC denoted with an * represent a statistically significant ROC curve. (**A**) ROC curves for biochemical values associated with all GH-secreting adenomas. (**B**) ROC curves for biochemical values associated with lactotroph adenomas. (**C**) ROC curves for biochemical values associated with corticotroph adenomas. (**D**) Biochemical lab test with corresponding predictive cut-off value, sensitivity, 1-specificity, and positive likelihood ratio.

Using ROC curves, we established biochemical cut-off values predictive of disease remission (Fig. 3). Preoperative IGF-1 index < 3.26 (Sensitivity (SENS) 0.833, Positive Likelihood Ratio (LR +) 1.46), POD1 GH < 1.37 ng/mL (SENS 0.813, LR + 7.46), and POM3 IGF-1 index < 1.26 (SENS 0.900, LR + 9.89) were established for all GH-secreting adenomas. Preoperative PRL index < 9.57 (SENS 0.845, LR + 2.14) and POD1 PRL index < 1.48 (SENS 0.897, LR + 4.875) were established for LAs. Finally, a POD1 ACTH < 25.5 pg/mL (SENS 0.838, LR + 2.52) and POD1 AM cortisol < 14 μg/dL (SENS 0.971, LR + 11.70) were established as biochemical cut-off values for CAs.

### Factors predictive of postoperative disease remission

**Figure 4:**
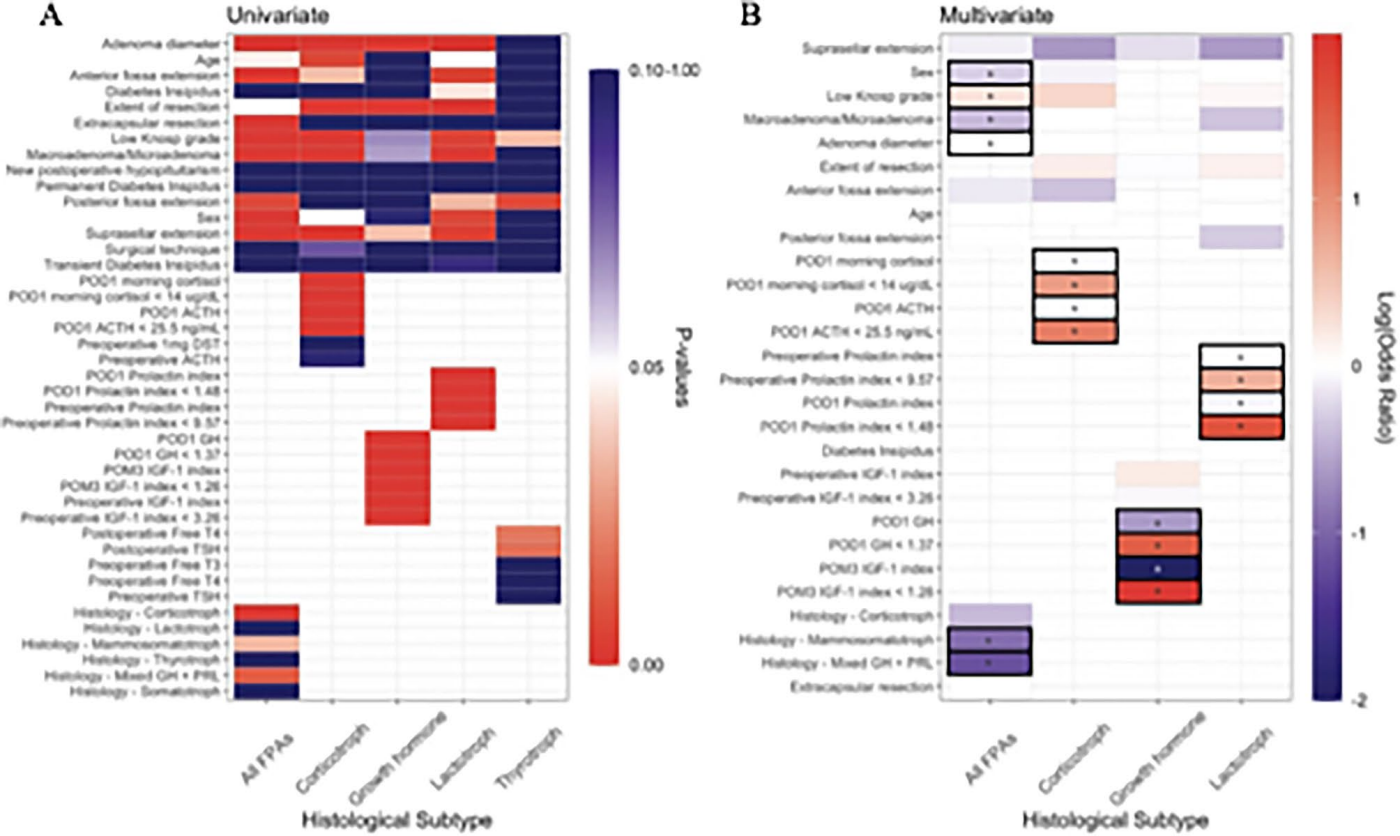
Univariate and multivariate regression analysis of factors predictive of postoperative disease remission. (**A**) Whole cohort and individual univariate analyses of variables associated with postoperative disease remission presented as tiled heatmaps. Red tiles represent a significant association with a p-value between 0 and 0.05. (**B**) Whole cohort and individual multivariate regression analyses of variables predictive of postoperative disease remission presented as tiled heatmaps. Variables included in multivariate models were found statistically significant in univariate analysis. Odds ratios are represented on a logarithmic scale. Red tiles represent a predictive factor of postoperative disease remission (OR > 1), while blue tiles represent a negative predictive factor for postoperative disease remission (OR < 1). Black-outlined boxes with asterisks identify statstatistically significant (p < 0.05) variables in multivariate regression analyses.

261 (66.6%) patients achieved postoperative disease remission. TAs had the highest rate of remission (N = 9, 90%), followed by CAs (N = 111, 77.6%), SAs (N = 60, 61.9%), LAs (N = 58, 60.4%), MSAs (N = 17, 56.7%), and mixed GH/PRL adenomas (N = 6, 37.5%). Univariate analysis revealed age, sex, adenoma diameter, macroadenomas, GTR, low Knosp grade (0–2), extracapsular resection, MSAs, mixed GH/PRL, and CAs to have an association with postoperative disease remission (Fig. 4A). These variables were incorporated into multivariate binary logistic regression (Fig. 4B). Multivariate analysis found male sex (Odds ratio [OR] 0.507, 95% Confidence Interval (CI) 0.310–0.829), increasing tumor size (OR 0.963, 95% CI 0.933–0.995), macroadenomas (OR 0.381, 95% CI 0.212–0.685), MSAs (OR 0.113, 95% CI 0.013–0.977), and mixed GH/PRL adenomas (OR 0.067, 95% CI 0.007–0.665) to be negative predictors of postoperative disease remission. While low Knosp grade (Knosp 0–2) (OR 1.74, 95% CI 1.015–2.996) and GTR (OR 4.245, 95% 2.525–7.137) were positive predictors of postoperative disease remission. No other variables were associated with postoperative disease remission in this group of patients.

In adenoma multivariate subgroup analysis, we did not identify demographic, radiographic or operative variables predictive of postoperative disease remission (Fig. 4A and B). However, we did identify strong pre-and postoperative biochemical predictors: for CAs POD1 serum ACTH < 25.5 pg/mL (OR 7.647, 95% CI 2.461–23.763) and POD1 morning serum cortisol < 14 μg/dL (OR 14.577, 95% CI 4.025–52.790), for LAs preoperative PRL index < 9.57 (OR 5.079, 95% CI 1.212–21.279) and POD1 PRL index < 1.48 (OR 41.824, 95% CI 11.719–149.267), and for all GH-secreting adenomas PO3M IGF-1 < 1.34 (OR 95.615, 95% CI 18.928–483.010) and POD1 GH < 1.37 ng/mL (OR 31.9, 95% CI 6.298–161.585). Additionally, we performed separate multivariate subgroup analysis replacing the dichotomous biochemical cut-off predictors with their linear scaled continuous variables (supplementary table 2). Similarlly to the dichotomous biochemical cut-off predictors, we found: for CAs increasing POD1 serum ACTH (OR 0.916, 95% CI 0.868–0.967) and POD1 morning cortisol (OR 0.923, 95% CI 0.866–0.984), for LAs increasing preoperative PRL index (OR 0.944, 95% CI 0.902–0.987) and POD1 PRL index (OR 0.452, 95% CI 0.106–0.508), and for all GH-secreting adenomas increasing POM3 IGF-1 Index (OR 0.01, 95% CI 0.001–0.099) and POD1 GH (OR 0.232, 95% CI 0.106–0.508) to be predictive of negative predictors of postoperative disease remission.

### Pit-SCHEME score

**Figure 5:**
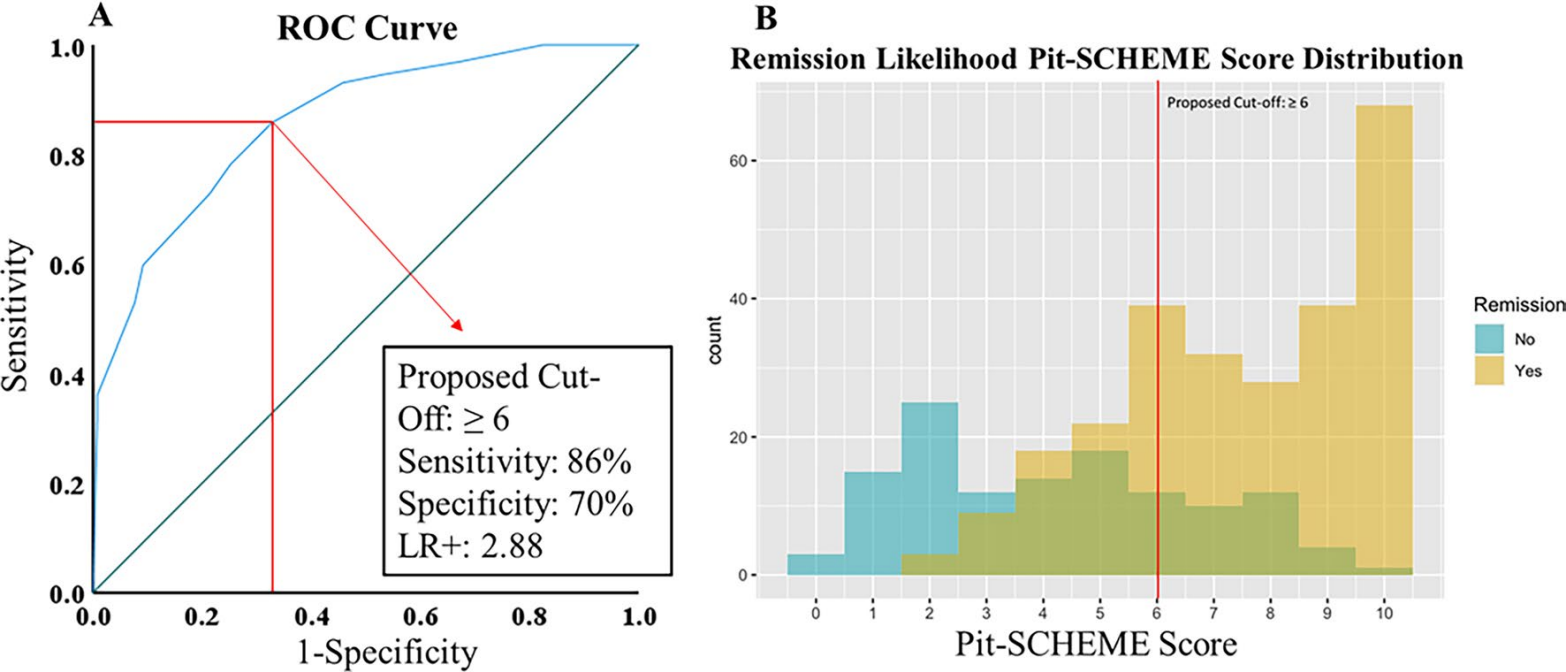
Analyzing the accuracy and score distribution of the Pit-SCHEME score. (**A**) ROC curve analyzing the prediction accuracy of the Pit-SCHEME score for postoperative disease remission. AUC: 0.858 (95% CI 0.820–0.895). A proposed cut-off score of ≥ 6 was selected for prediction of disease remission. (**B**) Score distribution of Pit-SCHEME score and corresponding rates of remission.

Variables found statistically significant on multivariate analyses were implemented to create our novel Pit-SCHEME (**S**ex, **C**avernous **S**inus Invasion, **H**istology, **E**xtent of **R**esection, **M**acro/Microadenoma, **E**ndocrinological values) score used to predict postoperative disease remission (Table 1). The score includes categories common to all histological subtypes of functional pituitary adenomas (sex, adenoma size, extent of resection, Knosp grade, histology). In other words, all patients are assigned a score in each of these categories. To account for the unique endocrinopathies associated with each histological subtype, (except for TAs) patients can be assigned additional points based on biochemical lab values related to their histological diagnosis. When devising the numerical portion of the score (i.e. point assignment) we aimed to not only create an accurate scoring system, but also an intuitive scoring system that is user friendly in the clinical setting. Thus, we designed the score to have limited variation in point assignments for each subcomponent (0 or 2/0 or 1) with a total score range of 0–10 (Table 1). For each category we approached point assignments using a holistic approach considering the magnitude of odds ratios, the percentage of our cohort with predictive factors that achieved disease remission, and the preexisting literature displaying the utility of the factor in predicting postoperative disease remission. The extent of resection, radiographic evidence of cavernous sinus invasion and macro/microadenoma categories were shown to have the highest and lowest OR magntiudes (4.245, 1.740, 0.381, respectively). Theferefore, these categories are award either zero (Knosp Grade > 3, macroadenoma, STR) or two points (Knosp Grade ≤ 2, microadenoma, GTR) (Table 1). In terms of patient sex, the score awards one point for females and zero points for males (Table 1). The subcomponent score for the sex category was based on the odds ratio for male sex (OR = 0.507) being greater than the other negative predictor (macroadenoma, OR = 0.381), and having relatively less evidence supporting postoperative disease remission in the preexisting literature when compared to variables like Knosp Grade, adenoma size and extent of resection^12–16^. In the histology category the following subtypes are represented: CA, LA, TA. SA, MSA, and mixed cell GH/PRL adenomas. Only GH and PRL co-secretion (MSA + mixed cell GH/PRL adenomas) subtypes were statistically significant in multivariate analysis (Fig. 4) and both displayed ORs < 1 (i.e., are negative predictors of postoperative disease remission). Given this, all GH and PRL co-secreting adenomas are given a score of 0 (Table 1). The other histological subtypes CA, LA, TA, and SA achieved greater than 60% remission, however none reached statistical significance. Thus, we decided to score these histologicall subtypes equally, that is they are all awarded one point in the Pit-SCHEME score (Table 1). The first five categories featuring common variables (sex, cavernous sinus invasion, histology, extent of resection, and microadenoma/macroadenoma) can produce a score ranging from 0 to 8. The last section of the Pit-SCHEME score involves endocrinological laboratory values and is segmented into three subcategories: lactotrophs, corticotrophs, and all GH-secreting adenomas (Table 1). Each subcategory contains two individual biochemical levels identified as predictive factors for postoperative disease remission (Fig. 4). A patient is rewarded one point for each biochemical level they are below (Table 1). Thus, according to individualized biochemical levels a patient can receive an additional 0–2 points. When all the categories are combined the Pit-SCHEME score has a range of 0–10 points (Table 1). When evaluating for multicollinearity between the individual subcomponents and the Pit-SCHEME score all variables displayed an independent relationship (met cut-off threshold of a tolerance > 0.1 and VIF < 10). The Pit-SCHEME score achieved a AUROC of 0.858 (95% CI 0.820–0.895) for all patients in our cohort. In terms of a predictive Pit-SCHEME cut-off score, we suggest a score ≥ 6 to be predictive of disease remission (SENS: 0.858, LR + 2.88) (Fig. 5). In the current cohort, patients with a Pit-SCHEME score ≥ 6 had a remission rate of 85.2%.

**Table 1:** Pit-SCHEME score for predicting disease remission of functional pituitary adenomas.

Measure		0 Points	1 Point	2 Points
**S**ex		Male	Female	
**C**avernous sinus invasion		Knosp Grade 3A, 3B, 4		Knosp Grade 0, 1, 2
**H**istology		Mammosomatotroph, Mixed cell GH/PRL adenomas	Corticotroph, Somatotrophs, Lactotrophs, Thyrotrophs	
**E**xtent of resection		Subtotal resection		Gross total resection
**M**acro/microadenoma		Macroadenoma (Size > 10 mm)		Microadenoma (Size ≤ 10 mm)
**E**ndocrinological Lab Values				
Lactotrophs	PreOp	Prolactin Index > 9.57	Prolactin Index < 9.57	
	POD1	Prolactin Index > 1.48	Prolactin Index < 1.48	
Corticotrophs	POD1	ACTH > 25.5 pg/mL	ACTH < 25.5 pg/mL	
		AM Cortisol > 14 μg/dL	AM Cortisol < 14 μg/dL	
All GH-secreting adenomas	POD1	GH > 1.37 ng/mL	GH < 1.37 ng/mL	
	POM3	IGF-1 Index > 1.26	IGF-1 Index < 1.26	

Postoperative Day 1 (POD1), Postoperative 3-month (POM3), Preoperative (PreOp), Prolactin (PRL), Growth Hormone (GH), Insulin-Growth Factor-1 (IGF-1), Adrenocorticotrophic Hormone (ACTH).

### Supervised machine learning

**Figure 6:**
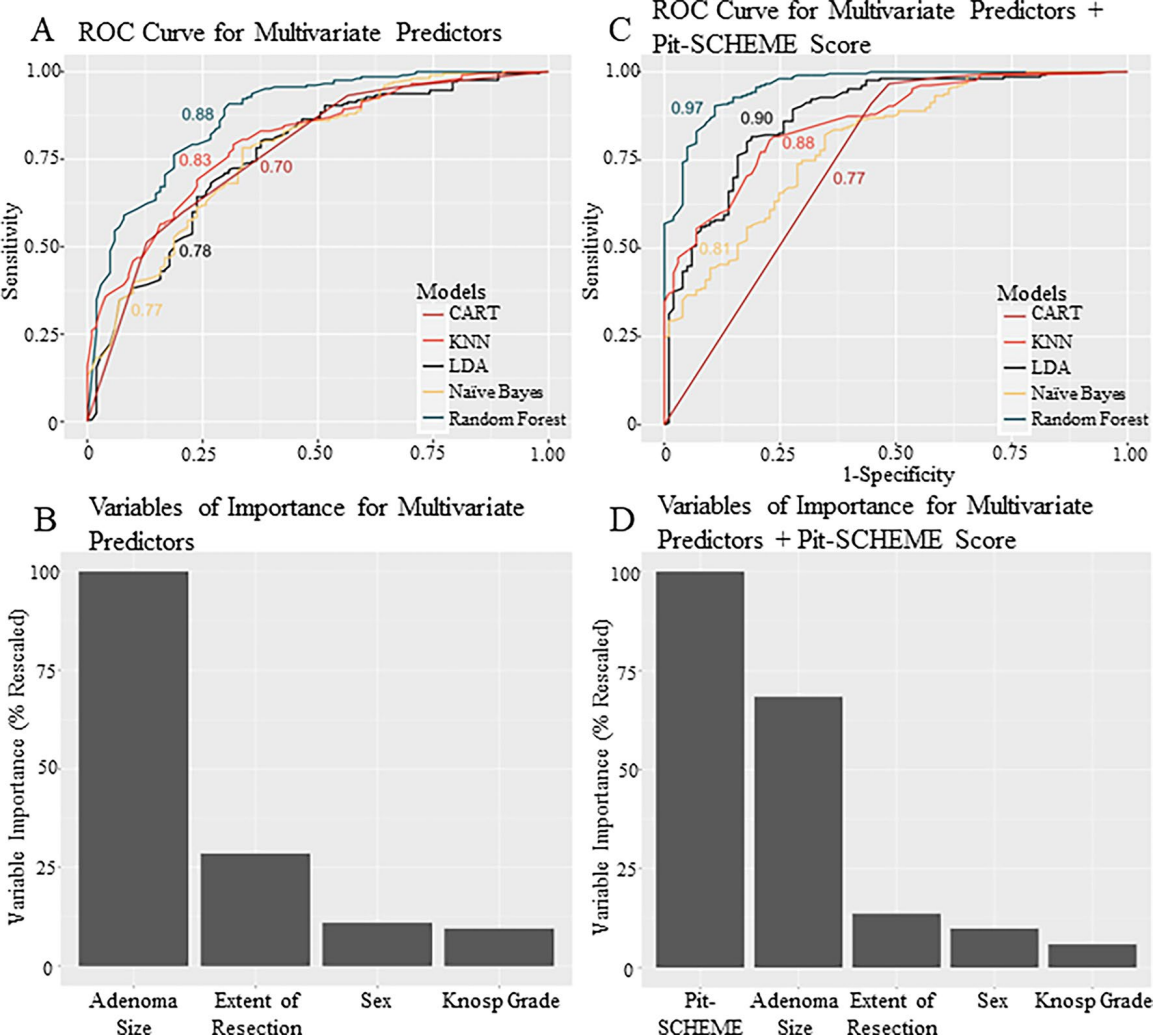
Using Supervised Machine Learning to validate the Pit-SCHEME score. (**A**) ROC curves for SML models including the six variables found predictive on multivariate analysis. CART and RF models achieved the highest accuracy. (**B**) Variable importance identified by SML models including the six variables found predictive on multivariate analysis. Adenoma size was the variable identified to have the greatest importance in predicting postoperative disease remission. (**C**) ROC curves for SML models including the Pit-SCHEME score. Model accuracy improved with inclusion of Pit-SCHEME score. RF displayed the highest accuracy. (**D**) Variable importance identified by SML models including Pit-SCHEME score. Pit-SCHEME score was identified as the most predictive variable for postoperative disease remission.

After training and cross-validation of the six SML models, they were tested on an independent testing set (Fig. 6 and Table 2). Without the Pit-SCHEME score, the CART and RF models achieved the highest accuracy at 72% (95% CI 60–81). The naïve Bayes model displayed the greatest sensitivity at 65% (95% CI 44–83); CART, kNM, and RF had the highest specificity at 79% (95% CI 65–89). Model performance was increased with the inclusion of the Pit-SCHEME score, with the RF model achieving the highest AUR-ROC, accuracy, and sensitivity at 0.970, 85% (95% CI 75–92), and 78% (95% CI 58–91), respectively. For all models, the inclusion of the Pit-SCHEME score improved model specificity. Without the the Pit-SCHEME score, the variable of most importance in the model was adenoma size. Subsequent addition of the Pit-SCHEME score, displayed it as the variable of most importance in our supervised machine learing models. Finally, a statistically significant difference in model prediction accuracy (without vs. with Pit-SCHEME score) for the LDA model (p = 0.0002), CART (p = 0.006), SVM (p < 0.0001) and RF (p < 0.0001).

**Table 2:** Supervised machine learning model performance.

Models with multivariate predictors						
Measurement (Value, 95% CI)	LDA	CART	kNN	SVM	Random Forest	Naïve Bayes
Accuracy	0.68 (0.56–0.78)	0.72 (0.60–0.81)	0.63 (0.51–0.74)	0.62 (0.51–0.74)	0.72 (0.60–0.81)	0.65 (0.54–0.76)
Sensitivity	0.62 (0.41–0.80)	0.58 (0.37–0.77)	0.42 (0.23–0.63)	0.50 (0.30–0.70)	0.58 (0.37–0.77)	0.65 (0.44–0.83)
Specificity	0.71 (0.57–0.83)	0.79 (0.65–0.89)	0.79 (0.65–0.89)	0.69 (0.55–0.81)	0.79 (0.65–0.89)	0.65 (0.51–0.78)
AUC-ROC	0.78	0.70	0.83	–	0.88	0.77

Models with multivariate predictors + Pit-SCHEME score						
Measurement (Value, 95% CI)	LDA	CART	kNN	SVM	Random Forest	Naïve Bayes
Accuracy	0.81 (0.71–0.89)	0.79 (0.68–0.87)	0.75 (0.63–0.84)	0.79 (0.68–0.87)	0.85 (0.75–0.92)	0.83 (0.72–0.90)
Sensitivity	0.74 (0.54–0.89)	0.56 (0.35–0.75)	0.56 (0.35–0.75)	0.56 (0.35–0.75)	0.78 (0.58–0.91)	0.63 (0.42–0.81)
Specificity	0.85 (0.72–0.94)	0.92 (0.80–0.98)	0.88 (0.75–0.95)	0.92 (0.80–0.98)	0.92 (0.80–0.98)	0.94 (0.83–0.99)
AUC-ROC	0.90	0.77	0.88	–	0.97	0.81

## Discussion

In the current study, postoperative biochemical remission for all FPAs was achieved in 66.6% (N = 261 of 392) of patients, which is similar to other studies investigating FPAs^12,17,18^. Our findings on multivariate analysis suggest GTR and low Knosp grades (0–2) are predictive of disease remission. CSI decreases the extent of resection which is associated with lower rates of biochemical remission^13–15^. The gold standard for determining true CSI is intraoperative visualization followed by histopathologic analysis of the medial wall of the cavernous sinus^14^. Due to variability of documentation of CSI in operative reports, we were limited to using preoperative radiologic Knosp grading criteria for CSI. Nevertheless, FPAs which do not exceed the intercarotid line (Knosp grade ≤ 2) on preoperative MRI are less likely to have true invasion into the cavernous sinus space^9,13,16^. Several studies have investigated the rate of CSI for FPA subtypes, and have found GH secreting adenomas to have the highest rate of CSI^19^. In our cohort, MSAs (43.7%) and mixed GH/PRL adenomas (46.7%) had the highest rate of radiographic evidence of CSI ( Knosp grade > 3), while CAs had the lowest rate (14%). Although none of these relationships reached statistical significance, suggesting in the current cohort, radiographic evidence of CSI is independent from a particular adenoma subtype. However, we acknowledge the findings of Mohyeldin et al.^19^ which supported that Knosp grade 2 SAs have a higher prevalence of true CSI when compared to other adenoma subtypes with the same Knosp grade. Thus, the rate of somatotrophic CSI in the current study may be underrepresented. Regardless, differences in the rate of remission between those with low vs high Knosp grades was not statistically significant for an individual FPA subtype (Fig. 4). Therefore, we suggest the preoperative Knosp grade is a predictive tool for postoperative disease remission and an appropriate measure in our scoring scale for all FPAs.

We identified that increased adenoma size was predictive of lack of biochemical remission following surgery, which is consistent with previously reported studies^12,20^. In the current study those with biochemical remission had a mean adenoma diameter of 12.08 mm (± 8.38) versus 19.71 mm (± 11.98) in those with continued disease. Moreover remission was achieved in 55.2% of patients with macroadenomas and 86.1% with microadenomas. The relationship between a larger adenoma size and decreased biochemical remission may be related to larger adenomas invading the parasellar space compromising critical neurovascular structures, limiting the extent of safe resection^12^.

Interestingly, we found gender as a predictive factor for postoperative disease remission, as females had a significantly higher rate of biochemical remission than males. Studies which simultaneously investigate multiple FPA subtypes have failed to identify the predictive value of gender for postoperative disease remission^17,18,21^. Although Chen et al.^22^ showed that male patients with pituitary adenomas had a worse overall survival, they did not establish if this was directly related to postoperative disease remission. Therefore, to our knowledge, we are the first to report gender as a predictive factor for remission of FPAs. In adenoma subgroup analysis, the sex difference in remission rate was noticed in all adenoma subgroups: LAs (73.1% female vs. 45.5%, male, p = 0.0006), CAs (80.7% female vs. 62.5% male, p = 0.030), and all GH-secreting adenomas (63.3% female vs. 49.1% male, p = 0.050). Our observation within the LAs population coincide with previous studies which found a higher rate of residual adenoma following TSS with subsequent lower rates of biochemical remission in men^23–25^. However, the LA cohort in Liu et al.^24^ was comprised entirely of male patients and all conclusions were drawn by comparisons to a nonfunctional adenoma subtype. Additionally, the gender differences established in Akin et al.^25^ were from a cohort comprosing LAs as well as other PRL co-secreting adenomas and thus not completely applicable to only LAs. Our results are most in line with Yoo et al.^23^ who found a similar association between remission rates and gender in a smaller cohort of 78 patients with LAs. Although these gender related differences in LAs are known, they are poorly understood. The lower rate of remission in men with LAs may be related to gender related tumor size, a higher rate of CSI and suprasellar extension, a poorer response to medical therapy, and a higher rate of cellular atypia, which cumulatively suggest a more aggressive phenotype in men when compared to women^23,26–28^. Ultimately, future studies are required to investigate the underlying association between disease remission and gender for all FPAs, particularly LAs.

On multivariate analyses we identified each adenoma subgroup (CAs, all GH-secreting adenomas, LAs) to have two statistically significant predictive biochemical cut-off values. For CAs, 92.9% of patients with a POD1 serum ACTH < 25.5 pg/mL and 89.7% with a POD1 morning cortisol < 14 μg/dL achieved disease remission. However, both values are higher than the commonly used cut-off values for CD remission (postoperative cortisol levels < 2 or < 5 μg/dL)^29–31^. Fewer studies have analyzed the predictive value of postoperative plasma ACTH for CD remission, with reported cut-off values including, < 34 pg/mL and < 10–20 pg/mL^29,32^. Nevertheless, these established cut-off values have a limitation as they are associated with the prediction of early disease remission (typically within first 48 h postoperative), and do not account for the subgroup of patients (~ 5.6%) who will experience a delayed decline (up to 6–12 weeks) before reaching a state of hypocortisolism^29,31–33^. Interestingly Valassi et al.^33^, who analyzed 35 CD patients with delayed biochemical remission following TSS, reported a mean postoperative cortisol (14 μg/dL) and ACTH (25 pg/mL) values which mirror our predictive cut-off values. Thus, we suggest the higher predicitive cut-off values in our study account for overall CD remission (not limited to early remission) and serves as a predictive measure for the Cushing’s population as a whole.

Here, we found patients with acromegaly with a POD1 GH < 1.37 ng/mL had a remission rate of 90.3%. This predictive value is slightly higher than the reported GH cut-off of < 1 ng/mL, which is associated with long-term disease remission and decreased mortality in patients with acromegaly and SAs^34,35^. However, we analyzed GH-hypersecretion in a patient population including SAs, MSAs, and mixed GH/PRL adenomas, with the later two thought to be a more aggressive tumor subtype^36^. Moreover, Dehghani et al.^36^ showed that dual-staining GH/PRL secreting adenomas to have higher mean postoperative GH levels than GH only SAs (1.69 ng/mL vs 1.30 ng/mL). Thus it is plausible our combination of all-GH secreting adenomas into a single group may account for the slightly higher postoperative GH cut-off value reported in this study. When considering the predictive value of postoperative IGF-1 levels, it is known IGF-1 levels fluctuate during the immediate postoperative period, and tend to stabilize at least 3 months following surgery^37^. Therefore, we evaluated IGF-1 levels at the 3 month postoperative mark and identified a POM3 IGF-1 index < 1.26 (91.5% remission rate) to be predictive of postoperative disease remission. Interestingly, our results are similar to Shen et al.^38^ who found an POM3 IGF-1 index < 1.485 predicted late onset disease remission (> 3-months following surgery). Thus, we propose our predictive POM3 IGF-1 cut-off value is representative for patients with normal and delayed postoperative IGF-1 decline rates.

We identified a preoperative PRL index < 9.57 (76.6% remission rate) and POD1 PRL index < 1.48 (88.1% remission rate) to be predictive of disease remission in patients with LAs. Our predictive preoperative PRL index is similar to those previously reported in the literature (< 100–200 ng/dL)^20,39^. However, given our PRL index accounts for gender differences in PRL expression it may be more predictive for all LAs, regardless of gender. Our POD1 PRL index cut-off is similar to results found in Amar et al.^40^ who identified patients with lower PRL levels (< 10 ng/dL) in the early postoperative period to have higher rates of disease remission. However, the conclusions in Amar et al.^40^ were not drawn from multivariate analysis and therefore may be confounded by other variables. As a result, we report a novel postoperative PRL index cut-off value predictive of disease remission in LAs.

Ultimately, the goal of the current study was to develop a novel scoring system for the prediction of disease remission in all patients with FPAs. Historically, this has been a difficult challenge as each FPA subtype is associated with unique endocrinopathies making it a clinically diverse population as a whole^2,22,30^. We attempted to resolve this problem by creating a scoring system which has common demographic, radiographic, surgical, and histologic measures which are predictive for all FPAs, while accounting for the unique endocrinopathies by providing predictive biochemical measures for each FPA subtype. ROC analysis displayed the Pit-SCHEME to be proficient in discerning those with and without postoperative disease remission, with a Pit-SCHEME score ≥ 6 being an appropriate cut-off score (SEN: 0.858, LR + 2.88). Moreover, we observed an independent relationship between the Pit-SCHEME score and its individual subcomponents (supplementary table 3). Ultimately this suggests that the Pit-SCHEME score does not represent the simple summation of predictive variables, but rather a unique independent variable representing a synergistic effect of its individual components.

We further validated our novel score using the latest advances in applied predictive modeling via SML. Compared to traditional statistical modeling techniques, SML trains an algorithmic model to identify the most predictive factors of an outcome based on training on a dataset with labeled examples of an outcome to train and identify the most predictive factors^11^. In the current study, our SML algorithms identified the most predictive variable for disease remission to the Pituitary-SCHEME score, with a model accuracy, sensitivity, and specificity of 85%, 78% and 92%, respectively. Thus, we posit in our retrospective cohort of 393 patients with FPAs the Pit-SCHEME score to be the most accurate predictor of postoperative disease remission.

### Advantages and limitations

We acknowledge there are limitations present in our current study given the retrospective nature. Our cohort is comprised of patients who underwent TSS using both endoscopic and microscopic techniques. However, neither were associated with remission rates in our cohort (65.6% and 68.4%), suggesting in our cohort, visualization technique did not significantly impact postoperative remission. A biochemical limitation in the present study is the inclusion of different assays with different normal age- and sex- specific ranges for measurement of IGF-1 levels (liquid chromatography- mass spectrometry and immunoassays). We attempted to mitigate this limitation by using an IGF-1 index with the upper range of normal IGF-1 values for sex and age according to the assay used to derive the measurement. We were underpowered to determine predictive biochemical cut-off variables for TAs. Also, we excluded functional plurihumoral tumors which were not dual GH + prolactin adenomas. However, TAs and plurihumoral tumors are exceedingly rare, and our results are applicable to the overwhelming majority of patients diagnosed with an FPA. Finally, we acknowledge the wide 95% CI reported for the ORs of our predictive biochemical laboratory values. In binary logistic regression analysis the OR represents the multiplicative increase in the odds of having a particular outcome and relies on all the variables in the cross-tabulation. The 95% CI width, also known as the degree of uncertainty, represents the precision of the OR estimate and varies according to the sample size and heterogeneity of the sample participants^41^. Particularly, it is dependent on the smallest entry of a crosstabulation table. In the current study, the biochemical cut-off values were derived from sensitivies obtained from ROC curves, not median or mean biochemical levels. Using high sensitivies to define biochemical cut-off values ensures capturing a high rate of true positives (i.e., patients below the biochemical cut-off value who achive biochemical remission) and limits the amount of false negatives (i.e., patients above the biochemical cut-off value who achive disease remission). Therefore, the biochemical cut-off values, particulary the distribution within our patient cohort, is not uniformily heterogenous (for example POD1 morning cortisol < 14 μg/dL, 89.6% N = 104 achieved biochemical remession (true positives) and POD1 morning cortisol > 14 μg/dL, N = 7 achieved disease remission (false negatives)). For comparison, we provided in supplementary table 2 the multivariate results of our adenoma subgroup analysis implementing the biochemical laboratory values as linearly scaled variables, which display statistically significant ORs with narrower, more precise 95% CI. Ultimately, this suggests the biochemical lab values are heterogenously distributed throughout our cohort and the wide range 95% CI seen in our biochemical cutoff value ORs is likely a result of sample homegeniety due to the selection of biochemical cut-offs with high sensitivities for postoperative disease remission. Regardless, our study is statistically appropriate as we implemented multivariate analyses (which controls for confounding variables) to create a novel predictive scoring system for biochemical remission, which was further validated across six-independent SML algorithms. Therefore, we believe our findings provide novel clinical insights into the management of patients with FPA.

## Conclusions

FPAs are associated with endocrinopathies resulting in significant morbidity and increased mortality. To date, modeling postoperative disease remission has proved challenging due to the heterogonous population of FPAs. We attempted to overcome this challenge by creating a scoring system to predict postoperative disease remission. Using the results of multivariate analyses we developed our novel Pit-SCHEME score with a predictive cut-off value of ≥ 6 (SENS 86%, LR + 2.88). SML further validated and identified the Pit-SCHEME score as the most predictive variable for postoperative disease remission in our cohort. These results support the potential clinical utility of the Pit-SCHEME score and its prospective future for aiding in the perioperative management of patients with FPAs.

### Supplementary Information

Supplementary Tables.

## Data Availability

Raw data used to draw conclusions in this study will be made available without hesitation upon request to the corresponding author K.C.
