# Supplementary material for: Supervised machine learning to validate a novel scoring system for the prediction of disease remission of functional pituitary adenomas following transsphenoidal surgery
Source: Sci Rep. 2023 Sep 16;13:15409. doi: 10.1038/s41598-023-42157-3 (PMC10505180; doi:10.1038/s41598-023-42157-3)
Supplement: Supplementary file 1 — Supplementary Tables. [file 41598_2023_42157_MOESM1_ESM.pdf]

Supervised Machine Learning to Validate a Novel Scoring System for the Prediction of Disease Remission of Functional Pituitary Adenomas Following Transsphenoidal Surgery

Chase McKevitt[1*], Ellie Gabriel[1], Lina Marenco-Hillembrand[1], Andrea Otamendi-Lopez[1], Suren Jeevaratnam[1], Joao Paulo Almeida[1], Susan Samson[1,2], and Kaisorn L. Chaichana[1]

Affiliations:
[1]Department of Neurological Surgery, Mayo Clinic, 4500 San Pablo Road, Jacksonville, FL, 32224, USA
[2]Department of Medicine, Division of Endocrinology, Diabetes and Metabolism, Mayo Clinic, 4500 San Pablo Road, Jacksonville, FL, 32224, USA
*Corresponding Author: Chase McKevitt, chase.mckevitt@gmail.com

Supplementary Table 1: Characteristics of 392 patients with functional pituitary adenomas	
Demographics	
Age	44.89 (±15.15)
Sex	
Male	123 (31.4%)
Female	269 (68.6%)
Histology	
Corticotroph	143 (36.5%)
Lactotrophic	96 (24.5%)
Somatotrophic	97 (24.7%)
Mammosomatotrophic	30 (7.7%)
Mixed Growth Hormone + Prolactin Adenoma	16 (4.1%)
Thyrotrophic	10 (2.5%)
Radiographic Features	
Adenoma diameter (in mm)	12.50 (IQR: 7.33-19.00)
Macroadenoma	248 (63.3%)
Microadenoma	144 (36.7%)
Knosp Grade	
Low Knosp Grade (0, 1, 2)	286 (73.0%)
High Knosp Grade (3a, 3b, 4)	106 (27.0%)
Suprasellar Extension	145 (37.0%)
Anterior Fossa Extension	30 (7.7%)
Posterior Fossa Extension	7 (1.8%)
Operative Variables and Outcomes	
Endoscopic technique	259 (66.1%)
Microscopic technique	133 (33.9%)
Extracapsular resection	124 (31.6%)
Piecemeal resection	268 (68.4%)
Gross-total resection	284 (72.4%)
Sub-total resection	108 (27.6%)
Postoperative Complications	
Postoperative CSF-leak	24 (6.1%)
New onset hypopituitarism	44 (11.2%)
Diabetes insipidus	
Transient	54 (13.8%)
Permanent	12 (3.1%)
Vascular injury	1 (0.3%)
Cranial nerve injury	6 (1.5%)
**Preoperative Biochemical Levels**	
Corticotroph Adenomas	
ACTH	74.00 pg/mL (IQR: 45.00-105.00 pg/mL)
1mg DST	14.27 ug/dL (SD: ±8.41 ug/dL)
Lactotroph Adenomas	
Prolactin Index	5.05 (IQR: 2.00-19.43)
All GH-secreting Adenomas	
IGF-1 Index	2.78 (SD: ±1.04)
Thyrotroph Adenomas	
TSH	5.22 uU/mL (SD: ±3.48 uU/mL)
Free T4	2.05 ng/dL (SD: ±0.92 ng/dL)
Free T3	7.05 pg/mL (SD: ±2.96 pg/mL)
**Postoperative Biochemical Levels**	
Corticotroph Adenomas	
POD1 ACTH	21.50 pg/mL (IQR: 13.00-28.00 pg/mL)
POD1 morning cortisol	5.00 ug/dL (IQR: 2.10-13.00 ug/dL)
Lactotroph Adenomas	
POD1 Prolactin Index	0.91 (IQR: 0.303-3.700)
All GH-secreting Adenomas	
POM3 IGF-1 Index	1.09 (IQR: 0.780-1.700)
POD1 GH	1.43 ng/mL (IQR: 0.7250-3.215 ng/mL)
Thyrotroph Adenomas	
Free T4	1.09 ng/dL (±0.40 ng/dL)
TSH	2.06 uU/mL (±2.04 uU/mL)
**Disease Remission**	
All FPAs	261 (66.6%)
Thyrotroph Adenomas	9 (90%)
Corticotroph Adenomas	111 (77.6%)
Somatotroph Adenomas	60 (61.9%)
Lactotroph Adenomas	58 (60.4%)
Mammosomatotroph Adenomas	17 (56.7%)
Mixed GH + PRL cell Adenomas	6 (37.5%)

Supplementary Table 2: Histological Subgroup Binary Logistic Regression Analysis using Linear Scaled Continuous Variables

Variable	p-value*	OR	95% CI
**Corticotroph Adenomas**			
Age	0.474	0.985	0.944-1.027
Size	0.897	1.007	0.902-1.125
Suprasellar extension	0.436	0.437	0.055-3.495
Low Knosp Grade (< 2)	0.716	1.487	0.176-12.586
POD1 ACTH	0.001	0.916	0.868-0.967
POD1 morning cortisol	0.014	0.923	0.866-0.984
**Lactotroph Adenomas**			
Age	0.280	0.982	0.949-1.015
Male Sex	0.402	1.768	0.466-6.705
Size	0.699	1.013	0.950-1.080
Suprasellar extension	0.167	0.475	0.166-1.365
Low Knosp Grade (<2)	0.927	1.063	0.289-3.905
Preoperative Prolactin Index	0.011	0.944	0.902-0.987
POD1 Prolactin Index	0.031	0.452	0.106-0.508
**All GH-secreting adenomas**			
Size	0.318	0.936	0.823-1.065
Male Sex	0.056	0.848	0.717-1.004
Suprasellar Extension	0.602	0.625	0.107-3.650
Low Knosp Grade (<2)	0.429	0.452	0.063-3.233
Preoperative IGF-1 Index	0.373	1.490	0.619-3.585
POM3 IGF-1 Index	<0.0001	0.01	0.001-0.099
POD1 GH	<0.0001	0.232	0.106-0.508

*p-value < 0.05 considered significant. Multivariate logistic regression of variables predictive of postoperative disease remission.

Supplementary Table 3: Multicollinearity Statistics

Variable	Tolerance*	VIF*
Adenoma diameter (micro/macroadenoma)	0.328	3.047
Knosp Grade	0.420	2.381
Extent of Resection	0.362	2.762
Sex	0.685	1.461
Histology	0.749	1.336
Biochemical values	0.834	1.199
Pit-SCHEME score	0.118	8.475

*Tolerance > 0.1 and VIF < 10 were thresholds for noncollinearity
